# An activity based model of grating and plaid adaptation in the human visual system

**DOI:** 10.1186/1471-2202-12-S1-P252

**Published:** 2011-07-18

**Authors:** Carl-Magnus Svensson, Stephen Coombes, Jonathan W Peirce

**Affiliations:** 1 School of Psychology, University of Nottingham, Nottingham, NG7 2RD, UK; 2 School of Mathematical Sciences, University of Nottingham, Nottingham, NG7 2RD, UK

## 

Adaptation effects to contrast, colour and motion are well-known in the visual system [1]. More recent psychophysics experiments have shown greater adaptation to compound stimuli than could be predicted by the sum of adaptations to their parts [2]. The apparent contrast of plaid stimulus (a pair of overlapping sinusoidal gratings) is less when observers have previously been exposed to that plaid than when they have been exposed only to its component gratings presented in a manner that equates the overall exposure. This effect suggests a neural mechanism that responds selectively to the compound stimulus. The differences in response after the adaptation show a strong dependence on the contrast of the probe, and the reason for this dependence is not clear.

We present a model that explores possible mechanisms that can mediate this type of selective adaptation to compounds. We use a network model [3,4] where each node represents a neuron (or population of neurons) with similar tuning characteristics. The first layer of the network represents the activity of V1 neurons, modeled using Wilson-Cowan-style dynamics. The responses are then fed on to subsequent layers, which also have feedback connections. Within each layer there are lateral connections that, for example, account for cross orientation inhibition as seen in V1. Each node dynamically adapts according to its recent activity.

We have explored a number of variants of this model, including different forms of linear and nonlinear adaptation dynamics, and different network topologies. The response properties of each network have been compared with the psychophysical findings. By doing this we isolate mechanisms capable of shaping the plaid-form-selective adaptation, particularly with respect to the novel contrast dependency found in our empirical studies.
